# Past-behavioural versus situational questions in a postgraduate admissions multiple mini-interview: a reliability and acceptability comparison

**DOI:** 10.1186/s12909-015-0361-y

**Published:** 2015-04-14

**Authors:** Hiroshi Yoshimura, Hidetaka Kitazono, Shigeki Fujitani, Junji Machi, Takuya Saiki, Yasuyuki Suzuki, Gominda Ponnamperuma

**Affiliations:** 1 Educational Committee, Prefectural Okinawa Nanbu and Children’s Medical Centre, Haebaru Town, Okinawa Prefecture Japan; 2 Educational Committee, Tokyo Bay Urayasu-Ichikawa Medical Centre, Urayasu City, Chiba Prefecture Japan; 3 Department of Surgery, University of Hawaii, John A. Burns School of Medicine, Honolulu, State of Hawaii USA; 4 Medical Education Development Centre, Faculty of Medicine, Gifu University, Gifu City, Gifu Prefecture Japan; 5 Faculty of Medicine, University of Colombo, Colombo, Western Province Sri Lanka

**Keywords:** Selection, Postgraduate training, Multiple mini-interview, Station interview format, Past-behavioural questions, Situational questions, Reliability, Acceptability, Multivariate generalisability analysis

## Abstract

**Background:**

The Multiple Mini-Interview (MMI) mostly uses ‘Situational’ Questions (SQs) as an interview format within a station, rather than ‘Past-Behavioural’ Questions (PBQs), which are most frequently adopted in traditional single-station personal interviews (SSPIs) for non-medical and medical selection. This study investigated reliability and acceptability of the postgraduate admissions MMI with PBQ and SQ interview formats within MMI stations.

**Methods:**

Twenty-six Japanese medical graduates, first completed the two-year national obligatory initial postgraduate clinical training programme and then applied to three specialty training programmes - internal medicine, general surgery, and emergency medicine - in a Japanese teaching hospital, where they underwent the Accreditation Council for Graduate Medical Education (ACGME)-competency-based MMI. This MMI contained five stations, with two examiners per station. In each station, a PBQ, and then an SQ were asked consecutively. PBQ and SQ interview formats were not separated into two different stations, or the order of questioning of PBQs and SQs in individual stations was not changed due to lack of space and experienced examiners. Reliability was analysed for the scores of these two MMI question types. Candidates and examiners were surveyed on this experience.

**Results:**

The PBQ and SQ formats had generalisability coefficients of 0.822 and 0.821, respectively. With one examiner per station, seven stations could produce a reliability of more than 0.80 in both PBQ and SQ formats. More than 60% of both candidates and examiners felt positive about the overall candidates’ ability. All participants liked the fairness of this MMI when compared with the previously experienced SSPI. SQs were perceived more favourable by candidates; in contrast, PBQs were perceived more relevant by examiners.

**Conclusions:**

Both PBQs and SQs are equally reliable and acceptable as station interview formats in the postgraduate admissions MMI. However, the use of the two formats within the same station, and with a fixed order, is not the best to maximise its utility as an admission test. Future studies are required to evaluate how best the SQs and PBQs should be combined as station interview formats to enhance reliability, feasibility, acceptability and predictive validity of the MMI.

## Background

The Multiple Mini-Interview (MMI) has been shown to be reliable [1-10] and acceptable [1,7-9,11-15] in not only undergraduate [1-4,11,12], but also postgraduate [5-10,13-15] medical selection in Canada [1,2,6,7,9,11,13,14], Australia [3], the UK [5,12,14], the US [4,8,15], and non-western countries [10]. As it overcomes ‘context specificity’ [1,16] through a wide sampling process, this selection instrument is considered more reliable than the Single-Station Personal Interview (SSPI), no matter how structured the latter may be [17,18]. A decade of research evidence suggests that a set of 10 to 12 stations with one examiner (interviewer) per station assessing candidates’ capabilities on multiple occasions (contexts) is proven to be reliable [1,6,7,19,20]. However, the structure of the MMI station per se varies from study to study, and from station to station, i.e., there is a range of the degree of: job analysis; developing the questions based on job analysis; standardisation of interview questions; standardisation of assessment format (rubrics of rating scales); and interviewer training [1-15,20]. Amongst those, as a station interview format, most studies have used the Situational Question (SQ) [21,22]: a question type of “what would you do in this situation?” combined with traditional SSPI questions: “tell me about yourself.” or “describe your strengths and weaknesses” [1-4,6-8,10]. Recently, within the nomenclature of ‘MMI’, station formats have been presented in a more complex manner including clinical knowledge tests [5], Situational Judgment Tests (SJT) [9], skills tests [9,15], role-playing with examiners [9,12,15], and interviews with SQs [1,5-7,9,12]. Some MMIs assess more than one candidate’s competency per station using assessment centre or selection centre principles [8,9,15,23-26]. Constructs assessed have also varied depending upon the availability of a job analysis with or without a set of nationally declared competencies, such as Canadian Medical Education Direction for Specialists (CanMEDS) Framework [6,26].

SSPIs are still ubiquitous in non-medical and medical selection [27]. As a part of the structuring process, both the Past Behavioural Question (PBQ) [22,27] and the SQ formats have been used widely [22,27]. PBQs asking “what did you do in the most recent past?” are derived from the idea that ‘the best predictor of job performance is the past behaviour’ [28]. Non-medical selection studies have demonstrated both PBQs and SQs in SSPIs have comparable reliability and acceptability, whereas PBQs have less fakability and higher predictive validity for high-complexity jobs than SQs [22,27]. In medical selection, especially in the postgraduate settings in the US, PBQ-based SSPIs have been adopted as the final selection tool of the residency matching process [29-35].

However, all the above studies on PBQs and SQs are related to SSPIs. To date, there are no reported studies on postgraduate admissions MMIs with stations of both PBQ and SQ formats. In this study, we investigated the research question: is there a difference in the reliability and acceptability of stations based on PBQs and SQs in a competency-based postgraduate admissions MMI, for Japanese medical graduates?

## Methods

This study received ethics approval from the Tokyo Bay Urayasu-Ichikawa Medical Centre’s (TBUIMC’s) Institutional Review Board, and Gifu University’s Research Ethics Board. The study procedure was fully explained and informed consent was obtained from all the participants.

### Settings and participants

TBUIMC is a Japanese general hospital, which newly introduced three specialty training programmes: internal medicine, surgery, and emergency medicine. To accomplish the trans-specialty mission of ‘fostering high-quality generalist physicians providing holistic patient care’, the educational committee of TBUIMC decided to introduce the Accreditation Council for Graduate Medical Education (ACGME) six general competencies [36] as educational outcomes. In 2013, the MMI took place at the partitioned TBUIMC conference room, in three separate weekends. Of the 26 candidates who applied for the TBUIMC programmes, 13, 10, and 3 were invited for the MMI on the first, the second, and the third day of the MMI, respectively.

Three separate days were set for candidates’ convenience, having better access to selection opportunities in TBUIMC; this facilitated the recruitment process. All candidates were Japanese medical graduates, whose level of training ranged from Post Graduate Year (PGY)-2 to PGY-4. They were either in the second year of, or had concluded the two-year National Obligatory Initial Postgraduate Clinical Training Programme (NOIPCTP), following their graduation from Japanese medical schools, and the Japanese National Licensure Examination [37]. A total of 18 examiners, including TBUIMC’s educational committee members (most of whom were US specialty board certified) and clinical supervisors, were all Japanese physicians in the aforementioned three specialties. All candidates, regardless of their applying specialties or the PGY level, were examined by all examiners, who were randomly allocated to the stations. All examiners stayed within the same station, on all three days.

### Intervention

To base stations on the competencies of the ACGME, except ‘medical knowledge’, 5 stations were created to assess one competency (domain) per station. Out of the 2 to 8 sub-domains in each competency [36], two sub-domains (one for the PBQ, and the other for the SQ) per station were selected so that one PBQ followed by one SQ was administered within the same station (Table 1). The same questions were asked from all candidates. Two examiners were assigned to one station and they alternated questioning roles. In PBQs, Situation-Task-Action-Result (STAR) approach was applied for guiding interviews [38]. In SQs, presenting a scenario with a dilemma and making the candidates describe what they would do, in a situation where the candidate had to choose between two or more mutually exclusive courses of action [21,22] were followed by structured probing [27]. Examiners were not allowed to probe independently. A sample of instructions to examiners for one of the stations is shown in Table 2.

**Table 1:** **Competencies (Domains), subdomains, and question types in the MMI stations**

Station number	Competency (Domain)	Sub-domain	Question format
Station 1	PCPS (IV.A. 5. a)*	(1)** Managing patient problems (treatment, health promotion)	PBQ
		(2)** Performing procedures competently	SQ
Station 2	PBLI (IV.A. 5. c)*	(8)** Educating others	PBQ
		(3)** Identifying and performing learning activities	SQ
Station 3	ICS (IV.A. 5. d)*	(1)** Communicating effectively with patients	PBQ
		(2)** Communicating effectively with physicians	SQ
Station 4	Pro (IV.A. 5. e)*	(4)** Being accountable to patients, society, and the profession	PBQ
		(2)** Responding patient needs that supersedes self-interest	SQ
Station 5	SBP (IV.A. 5. f)*	(5)** Working in interprofessional teams to enhance patient safety	PBQ
		(2)** Coordinating patient care within the health care system	SQ

PCPS: Patient Care and Procedural Skills, PBLI: Practice-Based Learning and Improvement, ICS: Interpersonal and Communication Skills, Pro: Professionalism, SBP: System-Based Practice.

PBQ: Past Behavioural Question, SQ: Situational Question.

*The Competency (Domain) number in ACGME Common Program Requirements [36].

**The Sub-domain number within the Competency (Domain) in ACGME Common Program Requirements [36].

**Table 2:** A sample of examiners’ interview guide (Station 3*)

Question type	Instruction
Question 1	✓Please do not ask any personal questions except brief and neutral greetings before starting.
PBQ	✓This question is to assess the ability of ‘communicating effectively with patients’. [IV. A. 5. d. (1)]
	✓The question to be initiated:
	Tell me about a difficult, cranky patient you had to take care of most recently during your NOIPCTP**.
	Please make your answer specific and concrete including the patient’s age, sex, problems, diagnosis, and management.
	✓Please use STAR*** approach as follows. Please do not make any other probing or follow-up questions.
	# What was the **S**ituation***? , and what was your **T**ask***?
	# What was your **A**ction***? How did you behave to establish a better relationship?
	# What was the **R**esult***? How did you obtain feedback from the patient or your team members?
Question 2	✓This question is to assess the ability of ‘communicating effectively with physicians’. [IV. A. 5. d. (2)]
SQ	✓The scenario to be presented:
	You have been working as a Year-1 resident of your specialty for 6 months. You have set in well with your new training environment. During a regular morning round, your clinical supervisor disagrees with one of your patient’s management plans which you feel confident about. You feel that you have been more involved in the care of this patient than the supervisor, including an understanding about the patient’s socioeconomic background. This supervisor is enthusiastic about patient care and teaching trainees, but you know that he does not like to have his authority questioned. He also seems to be inflexible in his thinking. How would you handle this situation?
	✓Please use following probing****. Please do not make any other probing questions.
	# Why would you take that action?
	# Is there any possible alternative ways in case that your initial plans do not work?
	# What are the advantages and disadvantages amongst your approaches?

*This station is to assess Interpersonal and Communication Skills (ICS) amongst the ACGME competencies.

**NOIPCTP: National Obligatory Initial Postgraduate Clinical Training Programme [37].

***STAR approach as described by Bangerter [38]. **STAR** is an abbreviation for **S**ituation-**T**ask-**A**ction-**R**esult probing question sequence, as indicated by boldfaced letters.

****Structured probing in SQ interviews was described by Levashina [27].

✓ Detailed instruction items.

# Specific probing questions to be asked.

All candidates were fully informed about the MMI logistics in advance by e-mail, and on the MMI day orally. No information about the ‘competency sub-domains’ that would be measured in stations was provided to the candidates. Prior to the MMI, the examiners were totally blinded to the candidates’ background information. Examiners were instructed to keep the interview questions on track, and to minimise close rapport building with the candidates during the examination.

Two examiners per station independently rated each candidate. Each answer was scored based on three rating rubrics: communication skills; strength and certainty of the answer; and suitability for the programme. Five-point rating scales were used and all points on a scale were anchored with descriptors (Table 3).

**Table 3:** **Rating rubrics**

Rubrics	Definition	Scoring
**Communication skills**	This candidate exhibits cooperative behaviour within the session:	5 - All A to E are seen fully.
	A. Listening carefully and actively, not interrupting the examiner, and clarifying the meaning of questions asked if necessary	4 - All A to C and either D or E are seen.
	B. Clear messages with confidence, not talking too much or too little	3 - All A to C are seen, but neither D nor E is seen.
	C. Constructive, open-minded, and optimistic attitudes	
	D. Using calm and steady voice tone and not talking too fast	2 - Two amongst A, B, and C are seen.
	E. Using non-verbal communications: eye contact; gestures; and a relaxed open stance	1 - One amongst A, B, and C is seen.
		Red Flag - None of A, B, or C is seen.
**Strengths and Certainty of Answers**	The behaviour s/he presents is true, and can be visualized clearly as if you see a movie:	5 - All A to C are seen fully.
	A. Answering every single structured probing question appropriately.	4 - Two amongst A to C are seen fully.
	B. Providing you with concrete and specific description of his/her own behaviour	3 - Only one amongst A to C is seen fully.
	C. Realistic and flexible decision-making	
	Faking, or deceptive Impression Management (IM*) should be assessed as “Red Flags”: extensive image creation; image protection; and deceptive ingratiation	2 - Two or three amongst A to C are seen weakly.
		1 - Only one amongst A to C is seen weakly.
		Red Flag - Any of IM types is observed.
**Suitability for the programme**	Likelihood that this candidate fits the organisational educational ethos: raising the high quality generalist within the specialty. S/he is trainable to pay full attention to biomedical, psychosocial, behavioural, and populational aspects of the patient, being interested in any organ systems or any clinical problems.	5 - S/he can work with us even now, being a self- directed learner with light supervision.
		4 - S/he will be competent early during the training.
		3 - S/he is tolerably trainable with full supervision.
		2 - S/he needs strenuous effort to be competent.
		1 - You feel great difficulty to train him/her.

*IM is defined as a process by which people attempt to influence the images others form of them during social interaction [27].

All examiners spent a total of 4 hours on training: 90 minutes of lecture on principles of the MMI, constructs to be assessed in each station, rationale for ‘structuring’ of interviews, definitions and procedures of PBQs/SQs, structured assessment formats, individual scoring based on anchored rating scales, how to counter interviewer bias (e.g. halo, or similar-to-me effect), and logistics of the interview day; 30 minutes of interactive questions and answers thereafter; and two separate occasions of one-hour mutual role-playing sessions by all examiners.

On the MMI day, a group of candidates rotated through five, two-examiner stations, each lasting 10 minutes and consisting of 5 minutes for the PBQ and then 5 minutes for the SQ. There was a one-minute break between the stations. On all 3 MMI days, the session began at 9:00 am, and finished within the same morning depending on the number of candidates. To implement the selection procedure smoothly and uniformly on all 3 days, a combination of two examiners (a pair), for a given station was fixed. After completion of all MMI stations, each candidate met programme directors (not the MMI examiners) of applying specialties. This final 30-minute informal session was held for recruitment, rather than for selection purposes, as it provided detailed information about the programme and answers to candidates’ questions.

### Post-MMI surveys

At the end of the whole schedule, all candidates and examiners were asked to complete an anonymous brief quantitative and qualitative post-MMI survey. The survey items probed: the candidates’ satisfaction with the abilities that were assessed, and the examiners’ opinion about the accuracy of assessing these abilities based on the PBQ and the SQ formats, as well as based on the overall examination; adequacy of time for the both formats; comparison of easiness of answering or questioning both formats; and fairness of the MMI on the whole, compared to the previously experienced selection SSPI. All responses were recorded using a 4-point Likert scale, with 1, 2, 3, and 4 indicating disagree, rather more in disagreement, rather more in agreement, and agree, respectively. Space for free comments was added. Both candidates and examiners were informed that individual survey answers would be kept confidential, and survey results would never affect any selection decision.

### Data analyses

The MMI scores were analysed with mGENOVA software (Version. 2.1) for multivariate Generalisability (G) and Decision (D) studies. The multivariate model for each PBQ and SQ format was:

$$ \mathrm{c}\cdotp \times \left(\mathrm{e}\cdotp :\ \mathrm{s}\cdotp \right) $$

c: candidate, e: examiners, s: stations, •: ratings (the fixed facet)

The ratings were considered as a fixed effect, since the three rating rubrics were considered as the universe under consideration, and were used in all stations. Hence, the generalisation over ratings was not required.

As to the post-MMI survey, paired t-test with a p-value of 0.05, was used for comparisons between PBQs and SQs in terms of the effectiveness in expressing/assessing candidates’ abilities, and the easiness of questioning/answering. Free comments were qualitatively analysed.

## Results

The mean age of the 26 candidates was 28.9 years (range 26-33). Of the 26 candidates, 20 (77%) were male and 6 (23%) were female. The male/female distributions on the first, second, and third day were 10/3, 8/2, and 2/1, respectively. Twenty-one were PGY-2 trainees of the NOIPCTP and 5 had progressed beyond the PGY-2 level; i.e. already joining individual specialty training. The numbers of candidates applying for specialties of internal medicine, surgery, and emergency medicine were 11, 6, and 9, respectively. The mean scores for PBQs were 4.13 (Standard Deviation [SD] 0.33), 4.13 (SD 0.30), and 4.11 (SD was not calculated because only 3 candidates participated in the session) for the first, second, and third days, respectively; those for SQs were 4.08 (SD 0.24), 4.05 (SD 0.32), and 4.04 (SD not calculated) for the first, second and third days, respectively. The mean scores of males were 4.09 for PBQs and 4.10 for SQs; those of females were 4.13 for PBQs and 4.08 for SQs.

### Reliability

The variance estimates are presented in Table 4. The variance component for candidates was the largest source of variance (see the set of rows for ‘c’ in the ‘effect’ column). Within candidates, the variance for communication skills in both PBQs and SQs (0.07938; and 0.03904, in the PBQ and SQ columns, respectively, shown in the first row for ‘c’ in the ‘effect’ column, indicated in bold) was the least, when compared with the other two ratings (0.11112[PBQ] and 0.13619[SQ]; 0.12635[PBQ] and 0.17173[SQ], shown in the second and third rows for ‘c’ in the ‘effect’ column, respectively). This indicates that there is relatively small candidate variability in their ability in communication skills. The variance of candidate-station interaction (see the set of rows for ‘cs’ in the ’effect’ column) was the second largest, but was smaller than that of candidates themselves in both PBQs and SQs. The variance of stations (see the set of rows for ‘s’ in the ‘effect’ column) and the variance of examiners within stations (see the set of rows for ‘e:s’ in the ‘effect’ column) were relatively small, indicating that there was no substantial station difficulty variation, or inter-examiner variability (including the issue of stringency/leniency), achieved by intensive station structuring process comprising: an established competency framework; standardised question types; standardised assessment rubrics with anchored rating scales; two independent examiners per candidate; and intensive examiner training. All these relatively small variances (except the candidate variance), suggest that context specificity was greatly reduced not only by the number of the stations, but also by overall station structuring process. The multivariate G analyses demonstrated that the G-coefficient was 0.822 for PBQs, and 0.821 for SQs. The D-study indicated that seven stations, each manned by one examiner would provide acceptable reliability (Table 5).

**Table 4:** **Variance components in PBQ stations and SQ stations**

Effect	PBQ station variance components			SQ station variance components		
	Communication skills	Strengths and certainty of the answer	Suitability for the programme	Communication skills	Strengths and certainty of the answer	Suitability for the programme
c	**0.07938**	0.62001	0.54169	**0.03904**	0.24238	0.0148
	0.05823	**0.11112**	0.83229	0.01767	**0.13619**	0.71386
	0.05425	0.09862	**0.12635**	0.00121	0.10917	**0.17173**
**s**	0.00277			-0.00596		
	0.01335	0.03204		0.01873	0.02204	
	-0.00644	-0.00767	-0.00965	0.01717	0.00171	0.02427
**e:s**	0.004			0.01262		
	0.00046	-0.00523		-0.00677	0.01169	
	-0.00446	0.00262	0.00954	-0.01323	0.00185	0.09262
**cs**	0.09723			0.05788		
	-0.00469	0.09104		-0.03075	0.12412	
	0.03529	0.012	0.20196	0.01648	-0.02671	0.21227
**ce:s**	0.18831			0.23738		
	-0.012	0.28215		-0.00477	0.21138	
	0.00446	-0.02185	0.24046	0.02092	0.00969	0.18815

N.B. – The negative variances, as they are very small in magnitude, could be considered as zero.

c- Candidate, s- Station, e- Examiner, e:s- Examiners within Stations, cs- Candidates into Stations, ce:s- Candidates into Candidates within Stations, and random error.

Bold figures indicate representatives of the largest source of variance amongst the effects.

**Table 5:** **The Decision (D) study for the PBQ and the SQ station formats**

Number of stations	Number of examiners per station	G-coefficient of PBQs	G-coefficient of SQs
4	2	0.787	0.786
5	2	0.822	0.821
6	2	0.847	0.846
5	1	0.766	0.751
6	1	0.797	0.783
7	1	0.821	0.808
10	1	0.868	0.858

### Acceptability

All candidates and examiners responded to the survey. As demonstrated in Table 6, this MMI on the whole was reasonably acceptable for all participants. While the majority of candidates perceived SQs as what could assess the candidate abilities best, the examiners felt the same for PBQs. Similarly, for easiness of answering/questioning, while for the majority of candidates SQs appeared to be the better format, for the examiners it was PBQs. These findings were statistically significant. All participants accepted that the MMI was fairer than the previously experienced SSPI. The free comments indicated that 19 candidates (73%) and 14 examiners (78%) expressed that both PBQs and SQs should be included in the MMI.

**Table 6:** **Post-MMI surveys**

Questions		Scores			p-value (< 0.05)
		3 + 4 ^¶^ (%)	4(n)	Mean (SD)	
1. In general, the current MMI allowed me to	Express my own abilities accurately. (C)	16 (62)	7	2.8 (0.87)	
	Assess candidates’ abilities accurately. (E)	12 (67)	2	2.7 (0.67)	
2. The 1^st^ question* allowed me to	Express my abilities accurately. (C)	10 (38)	1	2.3 (0.61)	#
	Assess candidates’ abilities accurately. (E)	14 (78)	4	2.9 (0.53)	##
3. The 2^nd^ question** allowed me to	Express my abilities accurately. (C)	19 (73)	2	2.8 (0.49)	#
	Assess candidates’ abilities accurately. (E)	7 (39)	2	2.4 (0.70)	##
4. For the 1^st^ question*, I had sufficient time to	Present my ideas.(C)	26 (100)	17	3.7 (0.45)	
	Manage sessions. (E)	18 (100)	13	3.7 (0.40)	
5. For the 2^nd^ question**, I had sufficient time to	Present my ideas.(C)	26 (100)	22	3.8 (0.26)	
	Manage sessions. (E)	18 (100)	7	3.4 (0.48)	
6. I did not have any difficulties to	Answer the 1^st^ question*.(C)	10 (39)	6	2.2 (1.08)	###
	Ask the 1^st^ question*. (E)	13 (72)	10	3.2 (0.86)	####
7. I did not have any difficulties to	Answer the 2^nd^ question**. (C)	18 (69)	4	2.7 (0.68)	###
	Ask the 2^nd^ question**. (E)	6 (33)	2	2.2 (0.78)	####
8. The current MMI is fairer than the SSPI	As a candidate.	26 (100)	5	3.2 (0.31)	
	As an examiner.	18 (100)	3	3.2 (0.28)	

MMI: Multiple Mini-Interview SSPI: Single Station Personal Interview C: Candidates E: Examiners n: number.

SD: Standard Deviation.

3 + 4^¶^: the sum of the number of participants who score the mark of 3 or 4.

*Past Behavioural Question **Situational Question.

p < 0.05 was observed between each of the same two marks of #,##,###, and ####.

Candidates (*C*) - n = 26, Examiners (*E*) - n = 18.

## Discussion

This study provides evidence that the competency-based postgraduate admissions MMI, containing either PBQs or SQs, could achieve acceptable reliability with ‘five, two-examiner stations’ (actual setting) or ‘seven one-examiner stations’ (D-study interpretation). Both formats were moderately acceptable for both candidates and examiners. Hence, the PBQ format is as reliable and acceptable as the SQ format.

In healthcare professional selection, studies attempting manipulation of the interview structure are scarce. An inter-rater reliability of 0.81 was obtained in dental undergraduate selection SSPIs, structured with the use of: job analysis driven competency-based framework; either PBQs or SQs as interview question types; behaviourally anchored rating scales; and panel interviewers [39]. However, since this was based on the SSPI format, it could not have addressed ‘context specificity’ [1,16] as appropriately as the MMI. More recent reports demonstrated G-coefficients of 0.76 and 0.69 for an undergraduate MMI with ‘four, one-examiner stations’ using PBQs and SQs, respectively [40], and a G-coefficient of 0.70 for a postgraduate MMI with ‘six, one-examiner stations’ formatted with PBQs [41]. There is no reported investigation other than the present study, which compares PBQs with SQs as station interview formats in the postgraduate admissions MMI.

The current study suggests that less than 10 stations of the MMI with one examiner per station may be sufficiently reliable. In addition to the question format, other structuring processes may have contributed to this, e.g. basing stations on an established competency framework; minimising unnecessary rapport building between examiners and candidates; asking exactly the same questions from each candidate with planned probing; using three distinguishable rating rubrics; rating candidates on points anchored with detailed descriptors; and providing examiner training. These structuring efforts would help reduce the number of stations, especially where only limited examiner resources are available for a relatively smaller number of candidates.

As non-medical personnel selection studies have suggested [27], the highly structured nature of the station interview formats and other structuring efforts in the present study may be responsible for the positive but modest candidate and examiner reaction compared with previous studies [1,7-9,11-15]. Interestingly, this study also indicates contrasting acceptability for SQs and PBQs amongst candidates and examiners, i.e. SQs being more favourable for candidates as opposed to PBQs being more favourable for examiners. Of particular note, all participants admitted fairness of the current MMI and most expressed importance of using both SQs and PBQs. As to how best PBQs and SQs could be combined, the participant reactions could be used as a guide for generating a discussion on both question formats at a given level (undergraduate or postgraduate [foundation, specialty, or subspecialty]) of admissions MMIs in the future, as is being discussed in the area of SSPIs in non-medical personnel selection [27].

This study has several limitations and weaknesses. Apart from the small number of candidates and some variability of PGY levels, the main limitation of the present study is related to two characteristics of the station structure: the PBQ-then-SQ fixed sequence (i.e. non-randomness of the order of questioning); and the inclusion of two question types (PBQs and SQs) within the same station (i.e. non-independence of the PBQ and SQ scores, meaning both the PBQ and SQ scores for a given competency domain being marked by the same set of examiners). Ideally, the MMI should have been conducted with PBQ and SQ sequence being randomly selected for a given candidate within a given station. If such a procedure was followed, the question order could have been included as another variable in multivariate generalisability analysis. As is, the variability introduced by the non-randomness of the question order would be within the random error of ‘ce:s’ in Table 4. In terms of reliability of the ‘entire’ MMI (i.e. when both the PBQ and SQ formats are considered as a whole), it would have been ideal if the PBQs and SQs were set up as different stations to obtain a series of examiners’ independent judgements on candidate ability. However, the research question of this study was to find out whether there is a difference in the reliability of PBQ and SQ based question formats. Hence, in the current study design, the examiners and candidates for a given competency domain were held constant, with the only variability coming from the question format; i.e. the question format was the only variable that was allowed to vary. Since the PBQ format and the SQ formats were analysed separately, non-independence of scores (i.e. having both question formats within the same station) was not taken into account in the multivariate generalisability study. This is said, setting up independent stations for PBQ and SQ formats would have circumvented the issue of non-randomness of the question order. If, however, the PBQ and SQ questions were in separate stations, the examiners who examined a given competency domain using PBQ and SQ formats would be different. This, although would address the non-randomness of the PBQ-SQ question order, would introduce more variability in terms of the examiners assessing a given competency being not the same. With regard to acceptability, the answer to the first question (PBQ) could influence examiners’ impressions on the second question (SQ), i.e. this fixed sequence might affect both candidates’ and examiners’ perceptions. To minimise this effect, a PBQ and an SQ individually addressed two different competency sub-domains (but within the same main domain) per station and importance of independent assessment for two question types, even within the same station, was intensively emphasised in examiner training. Despite the effort, a series of completely independent judgements on sub-domains might not be obtained, and therefore, this could compromise the comparison of the degree of acceptability between the two types of questions. Statistically significant candidates’ preference for SQs might be due to the adaptation to the station session, since SQs were asked as the second question. Likewise, statistically significant examiners’ better feelings for PBQs might be due to an advantage of sustainability in attention or mental efficiency since PBQs were always used first. Such biases could have been only eliminated by random selection for order of the two questions within the same station. In the present study, the effect of the order of two question types within each station was not explored because a part of the data were not generated first by the SQ and then by the PBQ; instead, all were only generated first by the PBQ and then by the SQ.

As is always the case with Japanese postgraduate selection setting, the TBUIMC facility only yielded space for a few stations, whereas a total of 10 stations would have been required to assess 5 sub-domains by the design of one question (for one sub-domain) type per station, which yet, would yield more examiner variability than that of two question types at a time, for a given competency. Furthermore, the fixed order of questioning had to be adopted to simplify this MMI implementation, given that all candidates and examiners experienced the MMI for the first time. Two more concerns are as follows: since three MMI sessions were set for candidates’ convenience, there might be leakage of interview questions; participants might not feel secure because this study was conducted without piloting, despite the sensitive and summative nature of selection, and without prior experience in conducting MMIs in Japan.

## Conclusions

Both the PBQ and the SQ formats were similarly reliable and acceptable in a competency-based postgraduate admissions MMI with five, two-examiner or seven, one-examiner stations. Future research should explore how PBQs and SQs complement each other to obtain optimal reliability and acceptability. Finally, research should ultimately focus on predictive validity of the MMI with structured question types, i.e. whether PBQs and SQs are equally predictive of future performance of trainees at different levels of education.

## Abbreviations

MMI: Multiple mini-interview
SSPI: Single-Station Personal Interview
SQ: Situational question
SJT: Situational Judgment Test
CanMEDS: Canadian Medical Education Direction for Specialists
PBQ: Past Behavioural Question
TBUIMC: Tokyo Bay Urayasu-Ichikawa Medical Centre
ACGME: Accreditation Council for Graduate Medical Education
PGY: Post Graduate Year
NOIPCTP: National Obligatory Initial Postgraduate Clinical Training Programme
STAR: Situation-Task-Action-Result
G: Generalisability
D: Decision
SD: Standard Deviation
